# Patient Involvement in Health Care Decision Making: A Review

**DOI:** 10.5812/ircmj.12454

**Published:** 2014-01-05

**Authors:** Shaghayegh Vahdat, Leila Hamzehgardeshi, Somayeh Hessam, Zeinab Hamzehgardeshi

**Affiliations:** 1 Department of Health Service Administration, Science and Research Branch, Islamic Azad University, Shiraz, IR Iran; 2 Department of Midwifery, Mazandaran University of Medical Sciences, Sari, IR Iran

**Keywords:** Patient, Decision-Making, Health Care

## Abstract

**Background:**

Patient participation means involvement of the patient in decision making or expressing opinions about different treatment methods, which includes sharing information, feelings and signs and accepting health team instructions.

**Objectives:**

Given the importance of patient participation in healthcare decision making which empowers patients and improves services and health outcomes, this study was performed to review previous studies on patient participation in healthcare decision making.

**Materials and Methods:**

To prepare this narrative review article, researchers used general and specific search engines, as well as textbooks addressing this subject for an in-depth study of patient involvement in healthcare decision-making. As a result, 35 (out of 100 relevant) articles and also two books were selected for writing this review article.

**Results:**

Based on the review of articles and books, topics were divided into six general categories: definition of participation, importance of patient participation, factors influencing participation of patients in healthcare decisions, method of patient participation, tools for evaluating participation, and benefits and consequences of patient participation in health care decision-making.

**Conclusions:**

In most studies, factors influencing patient participation consisted of: factors associated with health care professionals such as doctor-patient relationship, recognition of patient’s knowledge, allocation of sufficient time for participation, and also factors related to patients such as having knowledge, physical and cognitive ability, and emotional connections, beliefs, values and their experiences in relation to health services.

## 1. Background

In various scientific fields, participation has different definitions. In sociology, participation means having a share in something, and benefiting from that share, or taking part in a group and thus collaborating with that group. In political sciences, participation means the following: if people do not feel distinct differences between different issues they are faced with, they do not care about them and consider their actions ineffective, and if people, due to limited knowledge and awareness, consider their participation ineffective, they prefer to have less participation (1). Investigation of studies on health care shows that the focus of interest has been on participation of patients in care and treatment decision making process, using such terminologies as “involvement”, “collaboration” and “partnership” of patients, “client”, “consumer”, and “user” (2). There are numerous views on the concept of patient participation. One view considers the person’s participation in treatment decisions made about his own health issues (3, 4). In another view, patient participation means involvement of the patient in sharing information, feelings and accepting doctors’ and nurses’ instructions (1).

Patients’ participation in decision making in health care and treatment is not a new area, but currently it has become a political necessity in many countries and health care systems around the world (3). A review of the literature reveals that participation of patients in health care has been associated with improved treatment outcomes. Moreover, this participation causes improved control of diabetes, better physical functioning in rheumatic diseases, enhanced patients' compliance with secondary preventive actions and improvement in health of patients with myocardial infarction (5-8). Emphasizing the importance of participation in decision making process motivates the service provider and the health care team to promote participation of patients in treatment decision making. These efforts include enhancement of patient access to multifaceted information providing systems and tools that help patients in decision making (9, 10). With enhanced patient participation, and considering patients as equal partners in healthcare decision making patients are encouraged to actively participate in their own treatment process and follow their treatment plan and thus a better health maintenance service would be provided (11).

## 2. Objectives

In this study we aimed to review previous studies on patients' participation in health care decisions.

## 3. Materials and Methods

General databases like Google Scholar, and specialized databases such as Medlib, Magiran, Iranmedex, SID, Scopus, Pubmed, Springer, and Science Direct, as well as textbooks addressing patients’ participation in healthcare were used. Keywords used to retrieve the relevant information from 1992 to 2012 were "patient engagement", "user involvement", "patient involvement", "patient participation", "decision making", "health care", "quantitative study", "qualitative study", "measurement", and "instrument".

### 3.1. Search Strategy

The search strategy for required articles was, for example, as follows: (i) one or more of text word terms relating to decision-making or patient involvement, and (ii) text word terms describing concept of patient participation. The text word terms were correspondingly modified for use in different databases. The key words which were used in this study were as follows: patient involvement; decision-making; health care; patient participation; shared decision-making. Limitation rule applied to the search strategy was the presence of the key words in the title and abstract. A large number of titles and abstracts were generated from these searchers. Two authors independently assessed this output and retrieved relevant articles for further assessment. The degree of agreement between the authors assessing the content was calculated using the kappa coefficient which was found to be adequate (kappa = 0.79).

#### 3.1.1. Inclusion Criteria

We included papers that described the involvement of patients, defined as “the active participation in health care decision-making, concept of patient participation, patient involvement in health care consultations, factors associated with patient involvement, impact of patient participation on clinical outcome, patient preferences for involvement, scales of patient involvement in health care decision-making.” We included all papers that described the effects of involving patients in health care decision-making. At the first step, 100 abstracts and 5 books were read by the authors. After this stage 30 irrelevant articles and 3 books were excluded. At the second step, 70 full text articles were read by the authors. At this stage 35 irrelevant articles were excluded. Finally, authors reviewed 35 papers and 2 books.

#### 3.1.2. Exclusion Criteria

We excluded papers that described patient involvement in other activities such as research, community development, health promotion, and the effects of involving patients in the planning and development of health care programs.

### 3.2. Categorization of Studies

Totally, one hundred articles and 5 books were searched in the databases. All articles were reviewed concerning the concept of patient participation in healthcare decisions, related factors and measuring tools. Eventually, 35 articles and two books were selected for the study. We reviewed 14 articles and 1 book from 1992 until 2005, 21 articles and 1 book from 2006 until 2012.

## 4. Results

Review of articles and books led to arrangement of topics into six general categories: definition of concept of participation; importance of patient participation; factors influencing participation of patients in health care decisions; method of patient participation process; patient participation tools and techniques; benefits and consequences of patient participation in health care decision-making. Results of some of the studies on participation of patients in healthcare decisions are presented in Table 1. 

**Table 1.** Selected Summary of Some Studies on Patients’ Participation in Healthcare Decisions

Country of Study	Year of Study	Number of Participants/Study Population	Results
**Scotland**	2008	13	Participation as cooperation to understand information, not just seeking information	
**Sweden**	2004	10	Participation as trusting, understanding, seeking and maintaining a sense of control	
**England**	2000	44	The relationship of patients’ involvement with underlying factors, type and severity of disease, and patient-specialist relationship	
**11 European countries**	2000	330	The relationship of degree of patients’ involvement with doctor-patient interaction, patients' desire to participate, patients' demographics (literacy, high mental agility)	
**Sweden**	2006	26	The relationship between patients' involvement with knowledge, mental, physical and emotional capacity	
**Australia**	2006	73	The effect of patient-doctor interaction (discussion, trust) and doctors' interpersonal skills on participation	
**Scotland**	2007	20	The relationship between patients’ involvement with factors of respectful and friendly behavior, non-judgmental approach, doctors’ attention to patients’ views, clear explanation by doctors	
**Sweden**	2006	900	The relationship between patients’ involvement and factors of provision of information and explaining it according to personal needs, staff acknowledgement of patients’ knowledge	

### 4.1. Definition of the Concept of Participation

In Oxford dictionary, the word “participation” has been defined as engagement and involvement. This word is derived from the Latin word “Participare”, which means sharing (1). Personal participation is participation of the person in his own health care decisions, and public participation is referred to active group participation or participation of a person as representative of the group in developing the health system’s policies and plans. In recent years, patients and community participation has increased and patients and public more widely engage into it. Considering that people’s participation is voluntary, and it will not be realized until the individual and collective benefits are obtained; therefore, clear explanation of directives and policies and mutual understanding of people and community of healthcare programs are considered rules of engagement and active participation of people, which will lead to long-term partnership of both sides (3, 4).

Many studies have emphasized the concept of patient participation. Forbat et al have defined participation as cooperation for understanding information, as opposed to merely searching for information (12) (Table 1). In Eldh study, it is considered as being trusting, understanding and preserving feeling of control and recognizing responsibility of oneself as a patient (13) (Table 1). 

In the Sahlsten study, patient participation in nursing care is defined as patient/nurse relationship, control through the nurse, sharing information and knowledge, active participation in consultation and physical activity (2). There may be differences in definition and understanding of people’s participation in different sectors of health sciences. It is recommended that experts engaged in health view the subject from three different perspectives; first, in the medical approach, health has been defined as absence of disease, and people’s participation is defined as carrying out whatever the doctor has ordered. The second view is a health planning approach in which health has been defined according to the World Health Organization’s definition, which is not merely absence of disease, but physical, mental, and social well-being of people. In this view, people’s participation is defined as cooperation for receiving health services through payment of money, equipment, or manpower. The third perspective is the social development approach in which health is defined as a human condition, and people’s participation as active involvement in decision making and response to programs. Usually a combination of these three approaches should be considered as true patient participation (14).

### 4.2. Importance of Participation

Since adoption of any policies or decisions associated with health and treatment services eventually affects patients’ lives, patient participation in health affairs and development of macro health policies are considered among people’s civil rights. Also adoption of such policies are considered a sign of adopting good moral values---and a manifestation of equity and accountability in many health care systems in developed countries. Planning and provision of patient-oriented health care services based on the opinions, needs, and preferences of patients, those that care for them, and the community, is a key challenge in the health systems of developed countries, and it is an essential element in enhancing and improving health care systems in order to gain public approval and confidence. This leads to provision of more appropriate and cost-effective services, and ultimately enhanced health outcomes, quality of life, and satisfaction of patients (15).

 Of the most important reasons for the reforms in health care systems in developed countries in the last ten years are changes in people’s values, beliefs, and attitudes in respect of changes in community expectations, changes in patterns of diseases, increased life expectancy, and increasing emphasis on maximum level of health and quality of life, particularly in the last few years of life, which has been derived from the opinions of the public and the community. One of the most fundamental principles and policies of the new health care systems in these countries is valuing patients’ rights, and considering them as the axis for providing services, with special emphasis on the concept of patient and public participation and creating opportunities for all to share the decision on the method of receiving health care services.

 Ensuring that patient has appropriate information regarding the diagnosis and treatment is essential for providing safe and quality services. Providing appropriate information to the patients is an overwhelming process, but given its valuable outcomes in line with patients’ empowerment in making decisions after receiving the necessary information, its development has become a necessity. Surveying patients’ experiences and measuring results of the services provided from the viewpoints of patients would provide valuable information through which performance of service providers (from viewpoints of patients) can be compared. In this process, before and after every treatment procedure, the patient is asked about different aspects of quality of life such as level of pain, mental health, and ability and to move. In this way, efficacy of the treatment procedure, from the patient’s viewpoint, with regards to these three aspects of health is identified. The Patient Advice and Liaison Services (PALS) in the new health care system in England have been designed for providing more accountability to patients and clients. This, in fact, is a confidential service to ensure effective delivery of concerns and considerations of patients and service receivers to resolve problems with the least amount of bureaucracy involved (4, 16).

### 4.3. Factors Influencing Patient Participation in Health Care Decision-Making

#### 4.3.1. Patient-Related Factors

Demographic characteristics (17, 18); Personal characteristics (reads a lot, is mentally ok, can express himself) (3, 19); Level of acculturation(17), cultural knowledge (19) beliefs, values and practices concerning health and care (17); Having physical ability, cognitive and emotional relation with others (19, 20); Knowledge, beliefs, values and experiences in regard to mainstream health care services (17).

#### 4.3.2. Disease-Related Factors

Symptoms, stage, illness severity, experiences and meaning of illness (17, 18), health outcomes, health expectations, (3, 17-19), types of illnesses (3).

#### 4.3.3. Factors Related to Health Care Experts

The practitioner’s interpersonal communication skills (21) health care professional’s cultural competence, knowledge (knowing how to practice in a culturally informed and competent manner), beliefs and values (cultural, moral and professional), attitudes (respectful versus racist and ethnocentric toward ethnic minority patients), behavior (cultural skills and ability to form therapeutically effective relationships, engaging in cross-cultural communication, interviewing and assessing ethnic minority patients, addressing conflict, negotiating care, managing cases in a culturally informed and appropriate way) (17) health care professionals' knowledge and beliefs (18) features relating to the ethos and feel of healthcare encounters (welcoming; respectfulness; facilitation of patients’ contributions; and being non-judgmental) (22) patients’ relationship with professionals (3, 9), doctor listens and gives information (19), practitioners attending to patients’ views and patients feeling listened to; practitioners giving clear explanations based on their professional knowledge where patients understand these (22), considering the patient as an individual (23, 24), recognizing patients' knowledge (24), presence of a primary nurse/physician, encouragement of nurses and physicians to participate (19, 25), treating patients as equal partners in healthcare, nurses and physicians having enough time for patients(25).

#### 4.3.4. Factors Associated With Health Care Settings

Cultural appropriateness and competency of the system and organization, including: type and location of service, care processes, procedures and regulations, type and location of admission, (17) primary or secondary care (18)

#### 4.3.5. Factors Related to Health Provider Tasks

Cultural appropriateness and competency of the specific safety actions/behaviors (17); the required patient safety behavior might challenge clinicians' clinical abilities (18).

### 4.4. Patient Participation Process

It is highly important to know where to begin, whom to involve, in which processes and how they should be engaged. Since participation implies creating opportunities for people or groups to take part in decision making toward a specific objective, thus, any action in the area of patient and community participation should have a clear objective. In addition, participation process necessitates identifying individuals who are affected by the program. This process is known as stakeholder analysis. During this process, four critical questions are to be answered: Firstly, who will be affected by the program or project? Secondly, what kind of people worked in program in terms of time, money, sources or interests? Thirdly, if the process doesn’t invite individuals to participate, which group of people will question the organization? And fourthly, which people have authority for changes and improvement of program based on patients' and their health care provider experiences? The National Consumer Council has outlined “nine” principles for participation processes of patients and the public as follows: process must be able to affect change, process must be transparent, must have integrity and comprehensiveness, must be designed for a specific purpose, the process must include correct numbers and type of people, it must ensure people’s respect, give priority to participant’s debates, process must be reviewed and re-evaluated to improve services, the participating community must be well aware of the purpose of interactions (4). 

### 4.5. Patient Participation Tools for Evaluating the Participation

It is important to identify opportunities, and choose suitable tools and methods to evaluate people or community participation. The following qualitative and quantitative tools and techniques are used.

#### 4.5.1. Quantitative Methods

Such as surveys, although valuable, they only a look at snapshot of patients’ thoughts and beliefs, and require precise design and operational management to achieve valid results (4). For measuring patient participation, tools such as Patient Self-Advocacy Scale (PSAS) and Control Preferences Scale (CPS) are well known (26, 27). The PSAS tool comprises 12 items, and the following dimensions: Increased Assertiveness, Increased Illness Education, and Potential for Mindful Non-Adherence.

Information about the disease and healthcare enables people to exchange mutual information with healthcare providers. Education essentially helps enhance individual’s audacity and willingness to ask questions, and this will lead to participation in health care decisions. Patients’ heightened knowledge and audacity, and their better reasoning will result in non-compliance with health programs that are logically unacceptable to them (28). In the traditional compliance-gaining literature, patient non-adherence has been perceived by physicians as a form of deviance. However, Donovan and Blake asserted that non-adherence may not actually indicate deviance on the part of patients but instead may represent reasoned decision-making based on rational choices regarding lifestyle issues and treatments which are drawn from patient beliefs, responsibilities, and preferences(29); in this sense, non-adherence can be viewed as "mindful" rather than "mindless". Mindfulness is a state in which the individual consciously seeks environmental cues. Applying this concept to adherence to treatment regimens, we can view non-adherence as a strategic or mindful action when it is based on contextual considerations (e.g., the patient’s own health beliefs or life circumstances). Due to a desire for increased autonomy in their health care, activist patients are likely to have positive perceptions of and be willing to engage in instances of mindful non-adherence if they disagree with the efficacy of a physician’s treatment recommendations. In this case, instances of non-adherence behavior are not irresponsible or unreasonable but rather are carefully chosen actions based on the patients’ level of medical information about their disease and knowledge of their own personal health care needs and beliefs (26).

The Control Preferences Scale (CPS) tool has been considered for those patients that can have a role in decisions. The control priority of preferences is defined as a person’s level of control, when he wants to make a decision about his treatment (27). CPS consists of 5 cards (A-E), each depicting the text of different roles they have in decision making, with a spectrum from fully active to fully passive. Card A: (fully active), I prefer to have the final choice about my treatment. Card B: (semi-active), I prefer to make a decision after seriously considering the doctor’s opinions. Card C: (collaborative), I prefer to share the responsibility of decision making, regarding which treatment is best for me, with my doctor. Card D: (semi-passive), I prefer my doctor to decide after seriously considering my views. Card E: (fully passive), I prefer my doctor to decide about my treatment (30). CPS tool has been used in many studies. In the studies “it’s my body”: Does patient involvement in decision making reduce decision conflict? (31) and patient involvement in surgery treatment decision for breast cancer (32), using CPS, patient participations were 59% and 37.1%, respectively.

#### 4.5.2. Qualitative Methods

Such as focused group debates and interviews can also provide suitable data regarding patient’s values and beliefs. Many studies have used qualitative methods for collecting appropriate data, for example, Entwistle in his study used qualitative methods for collecting appropriate data (22). In a study, methods used to attract participants to patient participation programs included taking part in a group, workshop, meeting, seminar, group counseling, and individual interview (33).

### 4.6. Benefits and Consequences of Patient Participation in Health Care Decision-Making

The benefits of patient participation have been investigated in many studies. These benefits include: increased patient satisfaction and trust, higher patients’ quality of life, reduced patients’ anxiety and emotions, better understanding of personal requirements, more positive and direct professional’s communication with positive and lasting effects on health, patient empowerment and providing better patient health, receiving different opinions of patients about a common subject, planning and decision making improvements through combined opinions of patients, improvement of monitoring and evaluating services, better decision making due to access to different views, increased trust in services due to increased freedom, knowledge and transparency, a substantial opportunity for dealing with inequalities in health and access to services, encouragement of sense of independent responsibility, career promotion for most staff due to positive feedbacks, reduced possibility of patient dissatisfaction (11, 34, 35).

## 5. Discussion

In this study, 100 articles and 5 books were reviewed. Finally, 35 out of 100 articles and also two books were selected for writing this review article. Various distinct factors affecting patients’ involvement in healthcare decisions were considered by the researchers. Given the results, it can be argued that participation of patients is not merely for consultation, seeking opinions, or use of their actual and potential abilities, but also participation should result in better rehabilitation of patients. In this case, by acquiring knowledge, skills, and self-confidence, patients will be able to take care of their own health and manage to live life competently.

The current study findings showed that effective relationship of healthcare provider with patients is an important contributing factor of patient involvement in decision making. In addition, a literature review confirms that patients, in their journey through the healthcare system have the right to be treated respectfully and honestly, and where possible, be involved in their own healthcare decisions (36). Participation in decisions emphasizes priority of sensitive care, or helps when conditions of care are such that two or more treatment options are medically justified (9). For patients’ participation, mutual communication between treatment team and the patient is necessary, so that information and knowledge could be shared between them, giving the patient a sense of control and responsibility, and thus involving the patient in care activities (mental or physical), to benefit and rehabilitate from this involvement (13, 24, 37).

Consistent with the present findings, studies have shown that patient-physician relationship, discussion about treatment options, signs of the disease, course of disease, patient’s readiness to actively participate based on knowledge and physical ability, cognitive factors and emotional relationships, organizational factors, mutual trust, agreement between treatment team and patient, facilitating the grounds, giving responsibility to patient, and patient empowerment are factors associated with patient participation (1, 9, 18, 20).The present findings indicated that the patient participation in health care decision-making have several benefits. Similar to previous studies, this study showed that the added value of patients’ participation in healthcare includes receiving patient’s different points of view in relation to the same subject, improved services, better decisions due to access to different views, and career promotion for staff due to positive feedback (4). According to this study results and similar studies, emphasis on the importance of participation in decisions motivates the service provider and healthcare team to further increase patients’ participation in decisions (9, 10).

### 5.1. Strengths and Weaknesses

This review study on patient’s involvement in healthcare decisions is the first of its type conducted by Iranian researchers. In this article, comprehensive information about the concept of patient’s involvement and its importance, factors influencing involvement, and tools of involvement were presented. Both involvement and consultation have been regarded as two ends of a spectrum. Given that decisions associated with health services affect patients’ lives, thus, patients’ participation in health affairs is part of their rights, and symbolizes equity and responsiveness in many healthcare systems. This is what the present study is about, and what is meant by patients’ participation, which healthcare systems should take into consideration. In this study, factors affecting patients’ participation have been extensively emphasized. A few tools have also been discussed by the researchers, and finally, results and benefits of patients’ participation in healthcare process have been described. Study limitations included lack of access to full text version of some articles in some databases, which resulted in only partial use of these articles by the researchers.

### 5.2. Conclusion

In this study, considerations were given to the definition of concept of participation, importance of patient participation, factors influencing patient participation in health care decisions, methods of providing patient participation, patient participation tools and techniques, and benefits and outcomes of patient participation in health care decisions. In most studies, factors influencing patient participation, factors associated with health care professionals such as doctor-patient relationship, recognition of patient’s knowledge, allocation of sufficient time for participation, and also factors associated with patients such as having knowledge, physical and cognitive ability, and emotional connections, beliefs, values and their experiences have been in relation to health services. Patient participation in health care decisions is a sign of valuing humanity and individuality of the patient. Today, patient participation is regarded as a legal right of the patient as well as an international gold standard for healthcare systems, and health professionals strive for this standard. Patients must participate in decisions associated with planning, performance, and evaluation of healthcare.

### 5.3. Recommendations

Given that patient participation causes improved health outcomes, enhanced quality of life, and delivery of more appropriate and cost effective services, if patients are regarded as equal partners in healthcare, they would actively participate in their own health care process, and more carefully monitor their own care. Therefore, health professionals generally have a positive attitude toward patient participation, and consider this concept as a special privilege for themselves and the patients. Planning and providing patient-oriented healthcare, based on opinions, needs, and preferences of patients are recommended.
